# Supplementary material for: Optimization of differentiation and transcriptomic profile of THP-1 cells into macrophage by PMA
Source: PLoS One. 2023 Jul 17;18(7):e0286056. doi: 10.1371/journal.pone.0286056 (PMC10351730; doi:10.1371/journal.pone.0286056)
Supplement: S1 File — (ZIP) [file pone.0286056.s001.zip › supporting files/RNA-seq report.zip!/RNA-seq report/4_GOEnrichment_result/induce_vs_control/Enrich_bar/induce_vs_control.DEG_up_GO_Enriched_bar.pdf]

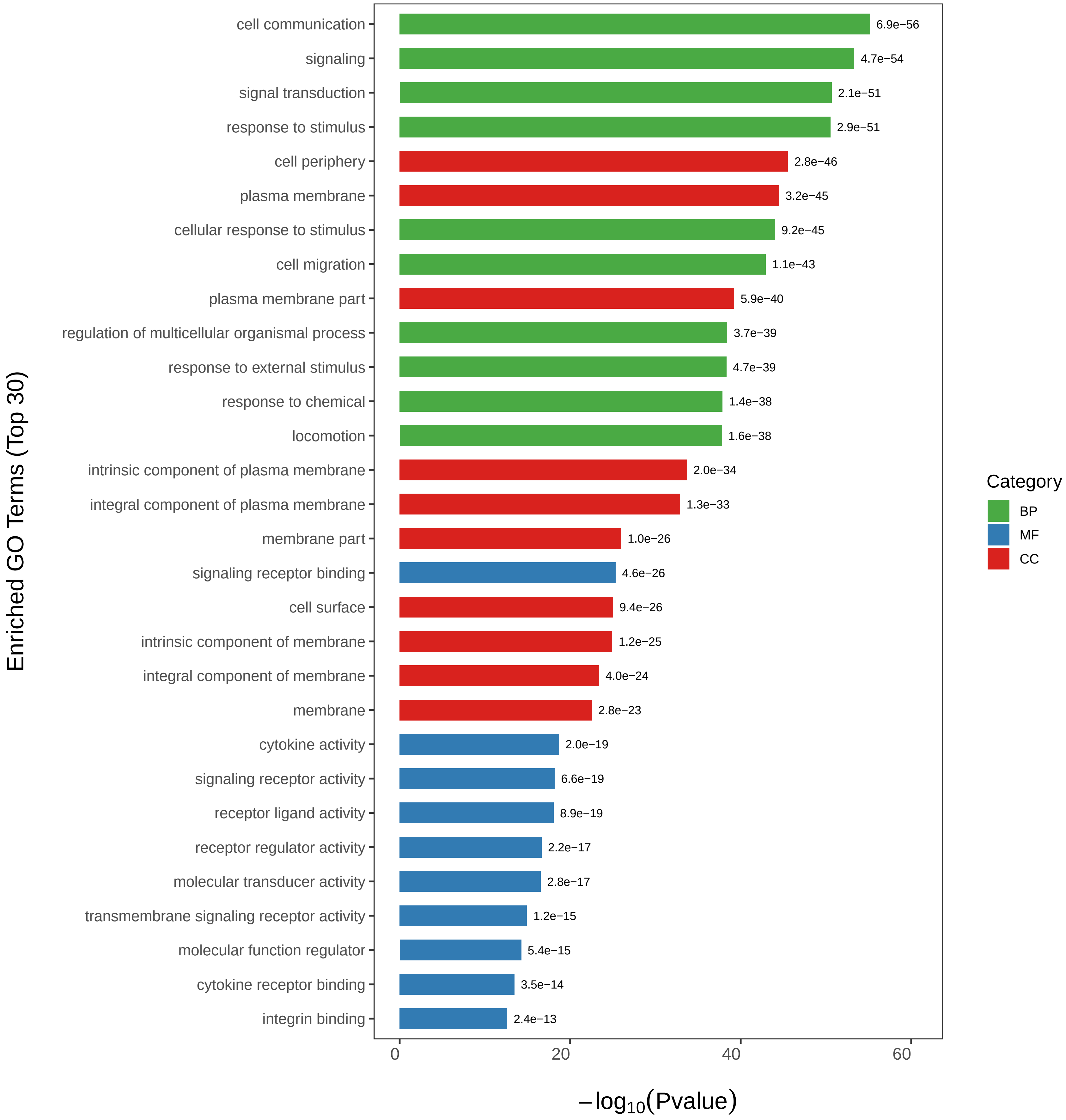

Enriched GO Terms (Top 30)
cell communication
signaling
signal transduction
response to stimulus
cell periphery
plasma membrane
cellular response to stimulus
cell migration
plasma membrane part
regulation of multicellular organismal process
response to external stimulus
response to chemical
locomotion
intrinsic component of plasma membrane
integral component of plasma membrane
membrane part
signaling receptor binding
cell surface
intrinsic component of membrane
integral component of membrane
membrane
cytokine activity
signaling receptor activity
receptor ligand activity
receptor regulator activity
molecular transducer activity
transmembrane signaling receptor activity
molecular function regulator
cytokine receptor binding
integrin binding
6.9e−56
4.7e−54
2.1e−51
2.9e−51
2.8e−46
3.2e−45
9.2e−45
1.1e−43
5.9e−40
3.7e−39
4.7e−39
1.4e−38
1.6e−38
2.0e−34
1.3e−33
1.0e−26
4.6e−26
9.4e−26
1.2e−25
4.0e−24
2.8e−23
2.0e−19
6.6e−19
8.9e−19
2.2e−17
2.8e−17
1.2e−15
5.4e−15
3.5e−14
2.4e−13
0
20
40
60
$-\log_{10}(\text{Pvalue})$
Category
BP
MF
CC
